# Analysis of sulfide signaling in rice highlights specific drought responses

**DOI:** 10.1093/jxb/erae249

**Published:** 2024-05-29

**Authors:** Jing Zhang, Angeles Aroca, Manuel Hervás, José A Navarro, Inmaculada Moreno, Yanjie Xie, Luis C Romero, Cecilia Gotor

**Affiliations:** Instituto de Bioquímica Vegetal y Fotosíntesis, Consejo Superior de Investigaciones Científicas and Universidad de Sevilla, Avenida Américo Vespucio, 49, 41092 Seville, Spain; Laboratory Center of Life Sciences, College of Life Sciences, Nanjing Agricultural University, Nanjing 210095, PR China; Instituto de Bioquímica Vegetal y Fotosíntesis, Consejo Superior de Investigaciones Científicas and Universidad de Sevilla, Avenida Américo Vespucio, 49, 41092 Seville, Spain; Instituto de Bioquímica Vegetal y Fotosíntesis, Consejo Superior de Investigaciones Científicas and Universidad de Sevilla, Avenida Américo Vespucio, 49, 41092 Seville, Spain; Instituto de Bioquímica Vegetal y Fotosíntesis, Consejo Superior de Investigaciones Científicas and Universidad de Sevilla, Avenida Américo Vespucio, 49, 41092 Seville, Spain; Instituto de Bioquímica Vegetal y Fotosíntesis, Consejo Superior de Investigaciones Científicas and Universidad de Sevilla, Avenida Américo Vespucio, 49, 41092 Seville, Spain; Laboratory Center of Life Sciences, College of Life Sciences, Nanjing Agricultural University, Nanjing 210095, PR China; Instituto de Bioquímica Vegetal y Fotosíntesis, Consejo Superior de Investigaciones Científicas and Universidad de Sevilla, Avenida Américo Vespucio, 49, 41092 Seville, Spain; Instituto de Bioquímica Vegetal y Fotosíntesis, Consejo Superior de Investigaciones Científicas and Universidad de Sevilla, Avenida Américo Vespucio, 49, 41092 Seville, Spain; University of Helsinki, Finland

**Keywords:** Comparative proteomics, drought tolerance, hydrogen sulfide, reactive oxygen species, stress response, water transport

## Abstract

Hydrogen sulfide regulates essential plant processes, including adaptation responses to stress situations, and the best characterized mechanism of action of sulfide consists of the post-translational modification of persulfidation. In this study, we reveal the first persulfidation proteome described in rice including 3443 different persulfidated proteins that participate in a broad range of biological processes and metabolic pathways. In addition, comparative proteomics revealed specific proteins involved in sulfide signaling during drought responses. Several proteins are involved in the maintenance of cellular redox homeostasis, the tricarboxylic acid cycle and energy-related pathways, and ion transmembrane transport and cellular water homeostasis, with the aquaporin family showing the highest differential levels of persulfidation. We revealed that water transport activity is regulated by sulfide which correlates with an increasing level of persulfidation of aquaporins. Our findings emphasize the impact of persulfidation on total ATP levels, fatty acid composition, levels of reactive oxygen species, antioxidant enzymatic activities, and relative water content. Interestingly, the role of persulfidation in aquaporin transport activity as an adaptation response in rice differs from current knowledge of Arabidopsis, which highlights the distinct role of sulfide in improving rice tolerance to drought.

## Teaser

Rice drought tolerance is enhanced by sulfide via persulfidation of specific proteins involved in different biological processes; the regulation of water transport associated with persulfidation of aquaporins is the most relevant.

## Introduction

Drought stress is a common adverse environmental condition that seriously restricts crop production and food security worldwide (Seki *et al.*, 2003). Plant adaptive responses to drought are coordinated by adjusting growth and developmental processes, as well as molecular and cellular activities. Drought stress triggers the production of reactive oxygen species (ROS) and the phytohormone abscisic acid (ABA), induces stomatal closure, and activates genes associated with signal transduction pathways (Qi *et al.*, 2018). It is also well documented that maintaining redox homeostasis and water status could improve plant tolerance to drought stress (Basu *et al.*, 2016; Zhu, 2016).

Hydrogen sulfide (H_2_S) has been recognized as a gaseous signaling molecule along with nitric oxide, carbon monoxide, and hydrogen peroxide (H_2_O_2_) in both animals and plants (Kimura, 2014; Aroca *et al.*, 2020; Zhang *et al.*, 2021). In recent decades, increasing attention has been given to the biological function of H_2_S in plants (Romero *et al.*, 2014). Numerous studies have suggested that H_2_S is involved in various developmental and stress response processes throughout the plant life cycle (Gotor *et al.*, 2019; Zhou *et al.*, 2021b). For example, H_2_S induces lateral root and adventitious root formation by modulating the expression of regulatory genes of the cell cycle (Mei *et al.*, 2017; Kou *et al.*, 2018). Pre-treatment with exogenous NaHS (a H_2_S donor) alleviates drought stress responses by re-establishing redox homeostasis and triggering ABA signaling in wheat and rice (Ma *et al.*, 2016; H. Zhou *et al.*, 2020).

Understanding the mechanism of action of H_2_S is of great importance in establishing the biological function of H_2_S in plants. The best studied mechanism of action of H_2_S in plants occurs through persulfidation, the oxidative post-translational modification of Cys protein residues (R-SH) by covalent addition of thiol groups to form persulfides (R-SSH) (Filipovic *et al.*, 2018). Persulfidation of proteins affects their structure, function, and subcellular distribution, thus providing a rapid and flexible mechanism for biological regulation during development and in response to environmental stimuli (Aroca *et al.*, 2017b; Filipovic *et al.*, 2018; Corpas *et al.*, 2021). Proteomic analyses of Arabidopsis protein persulfidation have revealed the regulation of protein function by H_2_S in diverse biological processes (Aroca *et al.*, 2015, 2017a; Laureano-Marin *et al.*, 2020; Jurado-Flores *et al.*, 2021, 2023b). Almost 13% of all annotated proteome proteins have been identified as targets for persulfidation in Arabidopsis. These proteins are involved in a wide range of biological functions, regulating important processes, such as primary metabolism, plant responses to stresses, growth and development, RNA translation, and protein degradation. Among these, ABA-regulated stomatal movement and autophagy have been well studied and are regulated by H_2_S-mediated persulfidation (Chen *et al.*, 2020; Laureano-Marin *et al.*, 2020; Shen *et al.*, 2020; Aroca *et al.*, 2021a; Zhou *et al.*, 2021a; Gotor *et al.*, 2022). In Arabidopsis, H_2_S-mediated persulfidation of key components in ABA signaling in guard cells and of autophagy-related proteins has been studied.

Rice is the most important food crop in the developing world and the staple food of more than half of the world’s population, but its grain yield is highly sensitive to environmental stresses (Yamaguchi and Blumwald, 2005; Seck *et al.*, 2012). However, knowledge regarding the functions and targets of persulfidation in rice plants is still lacking, and there are no previous data on its role in response to drought stress in this crop. The aim of this study was to investigate the molecular mechanisms by which H_2_S confers drought tolerance in rice plants, and, more importantly, to explore potential target rice proteins involved in the regulation and transduction of the H_2_S signal under both normal and drought stress conditions.

## Materials and methods

### Plant materials, growth conditions, and treatments

Rice (*Oryza sativa* L., *japonica*, Jsendra cultivar, Spain) (Reig-Valiente *et al.*, 2016) was used in this study. The seeds were surface-sterilized and germinated in distilled water for 2 d at 28 °C. The germinated seeds were sown in soil under a photoperiod of 12 h of white light (100 μmol m^–2^ s^–1^) at 28 °C and 12 h of darkness at 28 °C. For dehydration treatment, 25-day-old plants grown in soil under physiological conditions were divided into two batches with one batch irrigated with water and the other with 50 μM NaHS for an additional 10 d, and we replaced the NaHS solution every 2 d. After this period, each batch was subsequently divided into two new batches and subjected to water irrigation or drought for another 15 d. At the end of the full treatment, four different plant samples were obtained (Supplementary Fig. S1).

For protoplast preparation, the germinated seeds were grown hydroponically in plastic buckets with half-strength Murashige and Skoog medium for 15 d under the above-described growth conditions.

### Real-time reverse transcription–PCR analysis

Total RNA was isolated from 50-day-old rice leaves using TRIzol reagent (Invitrogen) according to the manufacturer’s instructions. Real-time quantitative reverse transcription–PCR (RT–qPCR) was performed using a Mastercycler ep® realplex real-time PCR system (Eppendorf) in a reaction mixture of 20 μl of SYBR-Green Premix (Vazyme) according to the manufacturer’s instructions. RT–qPCRs were carried out in a 20 µl volume, in 96-well blocks. The primers for the corresponding genes are listed in Supplementary Table S1. Expression levels of the genes are presented as values relative to their corresponding control samples after normalization to the levels of the *OsUBQ5* (Os06g0650100) and *OsACTIN1* (Os03g50890) transcripts.

### Dimedone-switch method and proteomics

The protocol was performed as described (Aroca *et al.*, 2022). Briefly, leaves from 50-day-old control or drought rice plants (Supplementary Fig. S1) were ground to a fine powder in liquid nitrogen. The frozen powder was transferred to an Eppendorf tube containing cold phosphate-buffered saline (PBS) lysis buffer (1× PBS pH 7.4, 2% SDS, and 1 mM EDTA), together with 5 mM 4-chloro-7-nitrobenzofurazan (Cl-NBF, SigmaAldrich) and 1% protease inhibitor, and incubated at 37 °C for 30 min, protected from light. A methanol/chloroform precipitation was performed to eliminate excess Cl-NBF, and the protein pellets obtained were washed with cold methanol, and dried. The dried proteins were dissolved in 50 mM PBS with 2% SDS, then incubated with 100 µM DCP-Bio1 (Kerafast) at 37 °C for 1.5 h, precipitated with methanol/chloroform, and finally dissolved in 50 mM PBS with 0.1% SDS. Proteins were incubated with Sera-Mag Magnetic Streptavidin beads (Cytiva) at 4 °C overnight with agitation. The microtubes containing the magnetic beads were located on a magnet and the beads separated from the supernatant, and were washed with 1× PBS supplemented with 0.001% Tween-20 several times. After washing, the beads were incubated with 2.25 M ammonium hydroxide overnight at room temperature, the final supernatants were transferred to fresh microtubes and neutralized with formic acid, and the beads were discarded. A total of 400 µg of proteins were trypsinized and analyzed by LC-MS/MS using a TIMS Tof Pro spectrometer (Bruker, Germany). The processed data were analyzed with the MQ (Max Quant) search engine and the label-free quantification (LFQ) was performed using PEAKS Studio (BSI, Canada). The search settings were carried out against a database of *O. sativa* (Uniprot/Swissprot) with precursor and fragment tolerances of 10 ppm and 0.2 Da, missed cleavages=2, modifications of lysine by NBF (mass shift: 163.0012), modifications of cysteine with hydrolyzed DCP-Bio1 (mass shift: 168.0786) or NBF (mass shift: 163.0012), and carbamidomethyl (C) and methionine oxidation (M) as variable modifications. Only proteins identified with at least two peptides at a false discovery rate (FDR) <1% were considered for further analysis. Protein abundances inferred from PEAKS were loaded onto a Perseus computational platform (Tyanova *et al.*, 2016), log2 transformed, and imputed. A *t*-test was used to address significant differences in protein abundances with each sample group under analysis.

The functional enrichment analysis and functional annotation of gene lists were performed using the web server Database for Annotation, Visualization, and Integrated Discovery (DAVID).

### Abscisic acid determination by UPLC‒MS/MS

Approximately 50 mg of frozen 50-day-old rice leaves were homogenized in 1.5 ml Eppendorf tubes (Eppendorf, Germany) for 2 min at maximum speed with a Retsch ball mill (MM400; Retsch). The metabolites were extracted from each aliquot in 0.8 ml of solvent mixture containing methyl-tert-butyl-ether:methanol (MTBE:MeOH, 3:1, v:v) cooled to –20 °C, with shaking for 30 min at 4 °C. The samples were sonicated in an ice-cooled bath for 15 min. An aliquot of 0.4 ml of acidified water (0.1% HCl) was added and thoroughly vortexed for 1 min. After that, the samples were kept on an orbital shaker for an additional 30 min at 4 °C. The samples were centrifuged at 10 000 *g* for 10 min at 4 °C. A fixed volume of the upper supernatant was transferred to a fresh tube and dried down using a Speed-Vac concentrator at room temperature. The dried pellets were resuspended in 100 μl of water:methanol (50:50) solution, and the resuspended samples were immediately subjected to UPLC‒ESI‒MS/MS hormonal analysis as described (Salem *et al.*, 2020). External ABA standard solutions were used for quantitation.

### Fatty acid determination

The lipids were extracted from 50 mg of 50-day-old rice leaves, ground to a fine powder in liquid nitrogen, and placed in a 1.5 ml Eppendorf tube with 120 µl of chloroform:methanol mixture (2:1 v:v). As an internal standard, 12 µg of heptadecanoic acid was added. The samples were vortexed and mixed with 180 µl of 0.7% (w/v) KCl. After centrifugation at 10 000 *g* for 5 min at room temperature, the lower lipid phase was collected and transferred to a glass tube. The sample was re-extracted with an additional 120 µl of chloroform:methanol mixture (2:1 v:v) and merged with the previous sample. Samples were evaporated to dryness under a stream of nitrogen gas. Fatty acid methyl esters were produced by transmethylation and analyzed by GLC (Garces and Mancha, 1993). The fatty acid composition was expressed in mol% of the different fatty acids and presented as the mean ±SE of three biological replicates and two technical replicates each.

The double bond index (DBI) was calculated from the mol% value using the formula: DBI=1×(mol% 16:1)+1×(mol% 18:1)+2×(mol% C18:2)+3×(mol% C18:3).

### ATP determination

The ATP in 50-day-old rice leaves was extracted following the boiling water method (Yang *et al.*, 2002) and was determined using a luminometric ATP assay. Briefly, 100 mg of frozen tissues were ground in liquid nitrogen, and homogenized in 500 μl of ice-cold distilled H_2_O, which was then boiled for 10 min in a boiling water bath. The boiled lysates were centrifuged at 13 000 *g* for 5 min at 4 °C and the supernatants were collected for ATP measurement using an ATP determination kit (ThermoFisher Scientific) following the manufacturer’s protocol.

### H_2_O_2_ determination

Fifty-day-old rice leaves (50 mg) were ground to a fine powder and H_2_O_2_ was quantified using the Amplex Red reagent (Thermo Fisher Scientific), as described (Jurado-Flores *et al.*, 2023a).

### Histochemical detection of ROS

The detection of H_2_O_2_ and O_2_^−^ was performed by 3,3-diaminobenzidine (DAB) and nitroblue tetrazolium (NBT) staining methods, respectively. The leaves from 50-day-old rice plants were excised and immersed in a 1% (w/v) solution of DAB (Sigma Aldrich) at pH 3.8 or a 0.1% (w/v) solution of NBT (Sigma Aldrich) in 50 mM potassium phosphate buffer (pH 7.8), containing 10 mM NaN_3_, vacuum-infiltrated for 5 min, and then incubated at room temperature for 8 h in the absence of light. The leaves were bleached by immersion in boiling 95% ethanol to visualize the spots and photographed.

### Determination of peroxidase enzyme activities

The total peroxidase (POD) activity was colorimetrically assayed with guaiacol as a substrate in 25-day-old rice seedlings pre-treated or not with 50 μM NaHS for 10 d and subjected to drought or normal irrigation. The reaction mixture contained 50 mM potassium phosphate buffer (pH 6.1), 0.25 mM H_2_O_2_, 6.3 mM guaiacol, and enzyme extract. The linear increase in absorption at 470 nm due to tetraguaiacol formation was followed for 2 min (extinction coefficient 26.6 mM^–1^ cm^–1^). For the determination of ascorbate peroxidase (APX) activity, the reaction mixture contained 50 mM potassium phosphate buffer (pH 7.0), 0.25 mM sodium ascorbate, and enzyme extract. The reaction was initiated by adding 5 mM H_2_O_2_, and ascorbate oxidation was determined by the absorbance decrease at 290 nm (extinction coefficient of 2.8 mM^–1^ cm^–1^).

### Relative water content determination

The leaf relative water content (RWC) was determined in 25-day-old rice seedlings pre-treated or not with 50 μM NaHS for 10 d and subjected to drought or normal irrigation as described (Ma *et al.*, 2016) using the formula: $RWC\left(\%\right)=\frac{FW-DW}{TW-DW}\times 100$, where FW is fresh weight, TW was turgid weight recorded by soaking the leaf sections in water for 24 h at 4 °C in the dark, and DW is the dry weight recorded after drying the samples in an oven at 70 °C.

### Protoplast preparation and water permeability assay

For protoplast preparation, the green tissues from the stem and sheath of 400–600 rice seedlings grown for 15 d were used. A bundle of rice plants (~30 seedlings) was cut together into ~0.5 mm strips with fresh razor blades without wounding. The strips were incubated in an enzyme solution (1.5% Cellulase RS, 0.4% Macerozyme R-10, 0.4 M mannitol, 20 mM KCl, 10 mM CaCl_2_, 0.1% BSA, and 20 mM MES at pH 5.7) into a vacuum desiccator for 30 min. After vacuum infiltration, the strips were further incubated for 4–5 h in the dark with gentle shaking (60–80 rpm). After the enzymatic digestion, protoplasts were released by filtering through 75 µm nylon meshes into round bottom tubes and centrifuged at 100 *g* for 3 min with a swinging bucket. The pelleted protoplasts were resuspended by gentle shaking in W5 solution (154 mM NaCl, 125 mM CaCl_2_, 5 mM KCl, and 2 mM MES at pH 5.7). After washing twice with W5 solution, the pellets were then resuspended in W5 at a concentration of 1 × 10^6^ cells ml^–1^, determined using a hematocytometer (Supplementary Fig. S2).

Water permeability of isolated rice protoplasts was measured by stopped-flow experiments at 20 °C by exciting at 450 nm and monitoring the net fluorescence intensity at 90 °C, using a µSFM-20 device fitted with a FC-08 cuvette (0.8 mm path length) and coupled to a MOS-450 spectrophotometer (Bio-Logic). For a single mix, 250 µl of protoplast suspension in W5 buffer was mixed with the same volume of W5 buffer containing 300 mM mannitol to make a hypertonic gradient in the flow cell. In addition, 250 µl of protoplast suspension in W5 buffer containing 500 mM mannitol was rapidly mixed with the same volume of W5 buffer containing 200 mM mannitol to make a hypotonic gradient in the flow cell. As a control, protoplasts in W5 or with 500 mM mannitol were mixed with the same volume of buffer W5 or with 500 mM mannitol, respectively, to compensate for any significant interference due to the stopped-flow mixing (not shown). The sulfide effect was determined in protoplast suspensions previously incubated for 5 min with 10 µM NaHS. The mannitol gradient causes osmotic water influx or efflux and protoplast swelling or shrinkage, and consequently changes in the internal chlorophyll fluorescence quenching (Wachlmayr *et al.*, 2022). Water permeability was followed by assuming a linear relationship between cell volume and the fluorescence signal (Wachlmayr *et al.*, 2022) and fitting the kinetic traces to a single exponential function to obtain the observed kinetic rate constants (*k*_obs_), using the Bio-Kine32 software package (Bio-Logic). Each kinetic trace was an average of 8–10 repeats.

### Inmunodetection of persulfidated aquaporins

Leaves from 50-day-old rice plants were ground in a mortar under liquid nitrogen and homogenized in 100 mM Tris–HCl, pH 7, 10 mM EDTA, 2% (w/v) SDS, and 6 M urea. After centrifugation at 10 000 *g* for 20 min, the supernatant was used as the protein extract. The total amount of protein was determined using the *DC* Protein Assay (Bio-Rad). The protein extract was divided into two batches, one of which was used for immunoblot analysis (input extract) and the second was subjected to the tag-switch method followed by immunoblot analysis (persulfidated extract).

The tag-switch method was performed as previously described (Aroca *et al.*, 2017a) with some modifications, consisting of the addition of 6 M urea in both the blocking and the CN-biotin labeling steps. The labeled proteins were purified by incubation with Sera-Mag Magnetic Streptavidin beads (Cytiva) overnight at 4 °C and subsequent elution from the beads with 2% SDS, 30 mM biotin, 50 mM phosphate, 100 mM NaCl, 6 M urea, and 2 M thiourea solution. Proteins of the input and the persulfidated extract were jointly separated using SDS–PAGE through the same 15% (w/v) polyacrylamide gel containing 6 M urea, and transferred to a nitrocellulose membrane. Proteins were detected with an antibody against aquaporin, plasma membrane intrinsic protein 1-3 (anti-PIP1;3, Agrisera, AS09 505), and horseradish peroxidase-conjugated anti-rabbit secondary antibodies (Bio-Rad, 170-6515), diluted 1:2000 and 1:30 000, respectively. As protein loading control of the input extract, the corresponding membrane portion was afterwards incubated with anti-tubulin (anti-Tub, Agrisera, AS10 680) diluted 1:5000. The immunodetected proteins were quantified using Image Labs software (Bio-Rad) and the level of each persulfidated protein was referred to its level in the input extract, in turn, relative to the tubulin protein level.

### Statistical analysis

All data are the means of at least three independent experiments. The data were subjected to ANOVA, and different letters in the figures indicate significant differences between treatments at *P*<0.05, according to Duncan’s multiple range test using the software package for statistical analysis (SPSS version 16, 2007).

## Results

### Exogenous application of sulfide improved rice drought tolerance

**Fig. 1.**
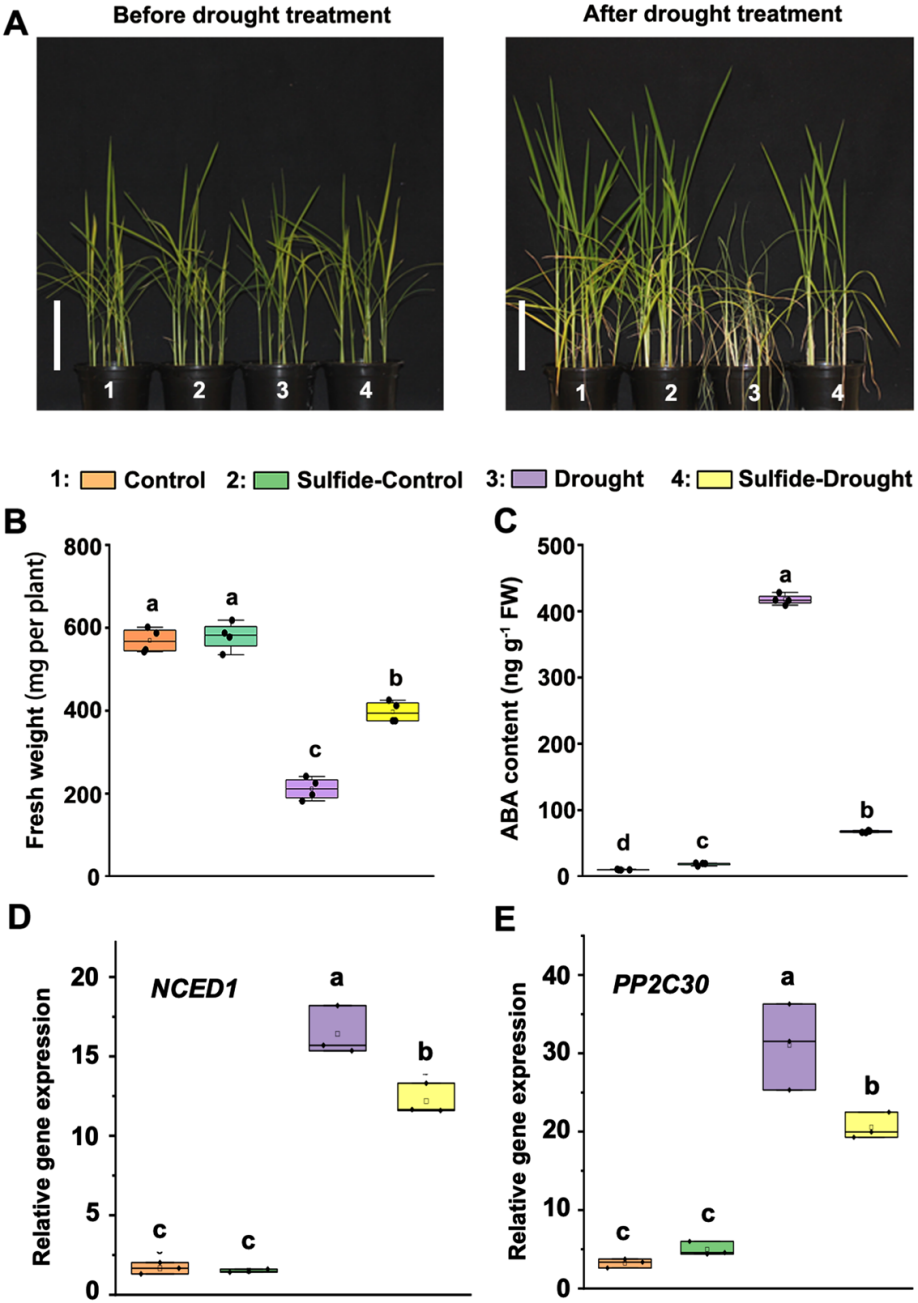
Exogenous application of NaHS alleviated drought stress in rice plants. Twenty-five-day-old rice plants were pre-treated or not with 50 μM NaHS for 10 d and then irrigation was withdrawn for 15 d. Afterwards, morphology (A), fresh weight (B), ABA content (C), and relative transcript levels of drought response genes, including *NCED1* (D) and *PP2C30* (E), were determined. Scale bar=10 cm. Data are means ±SD (*n*=4). The same letters indicate no significant differences. *P*<0.05 (Duncan’s multiple range tests).

Although a variety of studies have demonstrated the role of sulfide in inducing tolerance of plants to abiotic stress, such as drought, the mechanism of action of sulfide is beginning to be known only in *Arabidopsis thaliana* (Aroca *et al.*, 2021b; Zhang *et al.*, 2021; Zhou *et al.*, 2021b). To decipher the precise molecular mechanism underlying the function of sulfide in rice, we established an experimental system in which rice plants were subjected to drought conditions, as described in the Materials and methods (Supplementary Fig. S1). As shown in Fig. 1A, exogenous application of sulfide significantly improved rice growth performance under drought stress, as evidenced by the observation of a higher fresh weight of sulfide-pre-treated rice plants than under drought stress alone (Fig. 1B). Furthermore, we found that drought stress increased the level of ABA (Fig. 1C), as well as the relative levels of transcripts of drought response genes, including *9ʹ-cis-epoxycarotenoid dioxygenase 1* (*NCED1*, Fig. 1D) and *protein phosphatase type 2C 30* (*PP2C30*, Fig. 1E). However, pre-treatment with sulfide significantly reduced the effect of drought stress, as a very mild increase in ABA and the transcript levels of *NCED1/PP2C30* was observed (Fig.1C, E).

### Proteomic analyses provide insights into functionally enriched persulfidated proteins in rice

To gain insight into the protein persulfidation pattern in rice and to explore target proteins involved in the response to drought stress, a label-free quantitative proteomic approach based on MS combined with the dimedone-switch method (Aroca *et al.*, 2022) was applied to leaf samples under normal and drought stress conditions.

Protein samples from three biological replicates (independent pools) of leaf tissues from 50-day-old control or drought rice plants (nine plants per pool) (Supplementary Fig. S1) were isolated and subjected to the dimedone-switch procedure. The proteins eluted from the streptavidin beads were digested, and the peptide solutions were analyzed by LC-MS/MS. A total of 3324 and 3436 persulfidated proteins were identified in the control samples and in the drought-stressed samples, respectively. Among these proteins, 3317 were common in both control and drought samples, seven were only present in control samples, and 119 were only present in drought samples. In total, 3443 different proteins were identified as susceptible to persulfidation in rice, which is ~7.5% of the rice reference proteome (Supplementary Dataset S1). To our knowledge, this is the first proteomic analysis of persulfidation in cultivated plants. There is no doubt that this study will pave the way for a better understanding of the functional analysis of persulfidation in rice, unveiling a broad range of targets to be studied further.

**Fig. 2.**
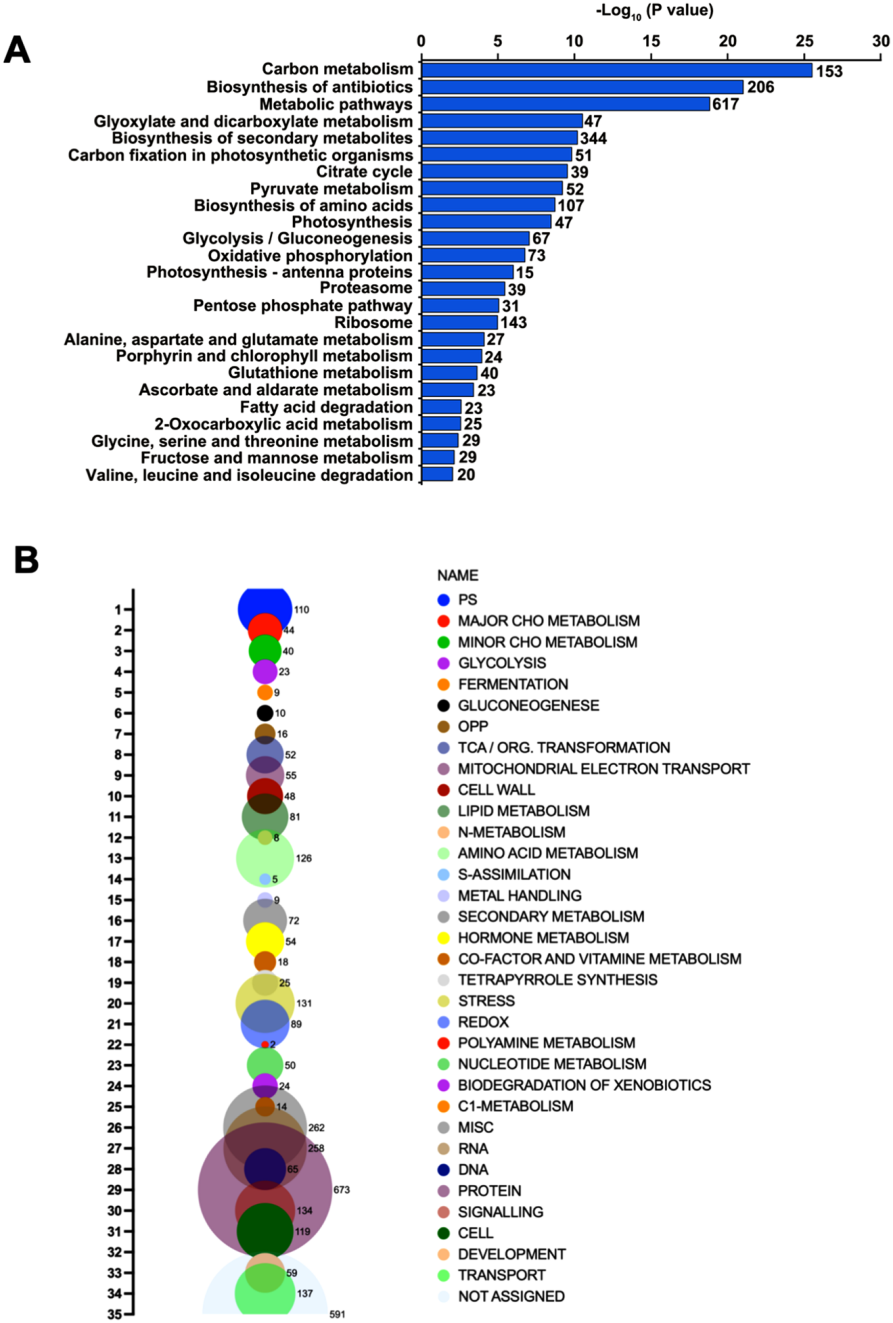
Functional classification of the total persulfidated proteins identified in 50-day-old rice plants. (A) KEGG pathway enrichment analysis of total persulfidated proteins. (B) Bubble plot of the functional classification of all the identified proteins according to the MapMan plant-specific database.

To assess the relevance of persulfidation in rice, the total identified proteins were analyzed by using bioinformatics resources from DAVID (*P*<0.05), and it was revealed that these persulfidated proteins covered most cellular functions. Gene ontology (GO) enrichment analysis based on the biological process category showed that a wide range of cellular and metabolic processes, including translation, protein folding, photosynthesis, tricarboxylic acid (TCA) cycle, glycolytic process, and cellular response to oxidative stress, were significantly enriched in persulfidated proteins (*P*<0.05; Supplementary Dataset S2). In addition, the functional annotation enrichment of the Kyoto Encyclopedia of Genes and Genomes (KEGG) pathway showed that the main categories in the KEGG pathway were carbon metabolism, biosynthesis of secondary metabolites, metabolic pathway, glyoxylate and dicarboxylate metabolism, carbon fixation in photosynthetic organisms, and others (Fig. 2A; Supplementary Dataset S2). In addition, ribosome functions and amino acid biosynthesis were found to be significantly enriched in the KEGG pathway, suggesting the potential role of protein persulfidation in the regulation of protein biosynthesis. Finally, the 3443 identified proteins were also analyzed based on their assigned functions and classified into 35 functional groups using the MapMan nomenclature developed for plant-specific pathways and processes (Klie and Nikoloski, 2012). The most abundant set corresponded to the general PROTEIN group, which included 18% of the total identified proteins, with proteins involved in protein degradation, synthesis, post-translational modification, and targeting (Fig. 2B). Two other important groups contained proteins involved in RNA and miscellaneous enzyme families, representing 6.9% and 7% of the total, respectively. Thus, the functional enrichment analysis of persulfidated proteins revealed the participation of a large number of these proteins in the regulation of important biological processes, such as carbon metabolism, photosynthesis, protein synthesis, responses to abiotic stresses, and RNA translation.

### Rice plants showed proteins differentially enriched in persulfidated fractions under drought stress

Although most of the persulfidated proteins were identified under both control and drought conditions, quantitative proteomics demonstrated different levels of persulfidation in these proteins, revealing a fine regulation by sulfide under drought stress. To identify specific persulfidated proteins that were relevant in H_2_S-dependent signaling during drought responses, we compared the persulfidation levels of the identified proteins by LFQ. The LFQ proteomic approach led to the quantification of 717 proteins that were differentially persulfidated in response to drought stress compared with control condition, with an FDR <0.05. For further analysis, seven persulfidated proteins that were exclusively identified under control and 119 only present under drought conditions were added. Therefore, a total of 843 proteins with differential levels of persulfidation were identified. Among these proteins, 635 were present only or were more persulfidated under drought stress (Supplementary Dataset S3), and 208 were only present or showed higher levels of persulfidation under control conditions (Supplementary Dataset S4).

Functional characterization and enrichment analysis of proteins that showed a higher level of persulfidation or were present only under drought stress were performed to reveal the regulatory role of H_2_S under this condition. At least 10 GO_biological processes (BP) and KEGG pathways were identified with >3-fold enrichment (Table 1). The protein group with the highest fold enrichment value corresponds to a subgroup of 15 proteins involved in the TCA cycle, such as oxoglutarate dehydrogenase, NAD-isocitrate dehydrogenase, and subunits of the succinate dehydrogenase complex. Other pathways and processes that showed a >3-fold enrichment were the glycolytic process and the amino acid and fatty acid degradation pathways, functionally related to the TCA cycle, as they supply metabolites to it through anaplerotic processes (Supplementary Fig. S3).

**Table 1.** Functional annotation by GO_biological function and KEGG pathway of the proteins only present or more persulfidated in drought samples and in control samples

Category	Term	Count	*P*-value	Fold enrichment	FDR
In drought					
GO_BP	Tricarboxylic acid cycle	15	1E-09	8.15	2.6E-07
GO_BP	Ion transmembrane transport	8	2E-04	6.31	1.8E-02
GO_BP	Cellular water homeostasis	8	2E-04	6.12	1.8E-02
GO_BP	Glycolytic process	11	2E-05	5.49	2.7E-03
GO_BP	Cytoplasmic translation	8	6E-04	5.29	3.8E-02
KEGG_PATHWAY	Valine, leucine, and isoleucine degradation	10	8E-04	3.85	9.0E-03
GO_BP	Translation	55	1E-16	3.59	5.3E-14
KEGG_PATHWAY	Pentose phosphate pathway	11	1E-03	3.26	1.1E-02
KEGG_PATHWAY	Fatty acid degradation	9	6E-03	3.13	3.3E-02
KEGG_PATHWAY	Glycine, serine, and threonine metabolism	12	2E-03	3.00	1.1E-02
In control					
GO_BP	Hydrogen peroxide catabolic process	18	4.6E-13	11.11	8.2E-11
GO_BP	Response to oxidative stress	17	8.9E-10	7.52	7.9E-08
KEGG_PATHWAY	Photosynthesis	7	1.8E-03	5.31	2.7E-02
KEGG_PATHWAY	Glutathione metabolism	7	1.2E-02	3.62	7.7E-02
KEGG_PATHWAY	Oxidative phosphorylation	9	3.3E-03	3.55	7.1E-02

The two other GO_BP classification terms that show a >6-fold enrichment correspond to ion transmembrane transport and cellular water homeostasis processes that both contain the same eight proteins of the aquaporin family, comprising six plasma membrane intrinsic proteins (PIPs) and two tonoplast intrinsic proteins (TIPs) (Table 2). The aquaporin PIP1-3 was only identified as persulfidated in drought samples, while the tonoplast protein TIP1-2 showed the largest significant change in persulfidation under drought stress, a 28-fold change.

**Table 2.** Quantified persulfidated proteins classified within the GO_biological process term ‘ion transmembrane transport’ and ‘cellular water homeostasis’

Accession	Gene name	Student *t*-test	*P*-value	Fold change drought/control
Os02g0823100	Aquaporin PIP 1-3	^*a*^	^*a*^	^*a*^
Os01g0975900	Aquaporin TIP1-2	–3.7426	2.01E-02	27.94
Os03g0146100	Aquaporin TIP1-1	–4.4663	1.11E-02	9.55
Os02g0666200	Aquaporin PIP1-1	–5.8649	4.22E-03	8.36
Os02g0629200	Aquaporin PIP2-2	–6.638	2.67E-03	6.02
Os09g0541000	Aquaporin PIP2-7	–3.5350	2.41E-02	3.92
Os04g0559700	Aquaporin PIP1-2	–13.996	1.51E-04	3.55
Os07g0448800	Aquaporin PIP2-1	–6.8503	2.38E-03	3.49

^*a*^ Only found in drought and therefore there is no associated *P*-value or fold change.

Regarding proteins with a lower level of persulfidation or absent under drought stress, functional enrichment analysis showed five GO_BP and KEGG pathways with a >3-fold significant enrichment (Table 1). The process with the highest enrichment corresponds to the H_2_O_2_ catabolic process that comprises 17 peroxidase enzymes, including cytosolic ascorbate peroxidase 1 (APX1) and chloroplastic thioredoxin M5 (Supplementary Table S2). In addition, the second enriched process corresponds to the response to oxidative stress, which includes 15 POD enzymes also included in the GO process mentioned above, as well as the methionine (R)-*S*-oxide reductase enzyme and the NADH dehydrogenase 1 alpha subunit. Related to oxidative stress, the glutathione metabolism pathway also shows a significant fold enrichment and comprises five glutathione *S*-transferase proteins, the APX1 enzyme, and the chloroplastic APX8. These findings could be indicative of the proposed role of persulfidation in protecting against protein overoxidation (Filipovic and Jovanovic, 2017; Vignane and Filipovic, 2023) and, in this case, under non-stress conditions, the antioxidative systems to maintain basal ROS levels were enriched. When the drought stress condition was imposed, other biological processes and pathways related to specific plant stress responses remained the most enriched.

### Persulfidation affects energy production, fatty acid composition, and antioxidant systems in response to drought stress

**Fig. 3.**
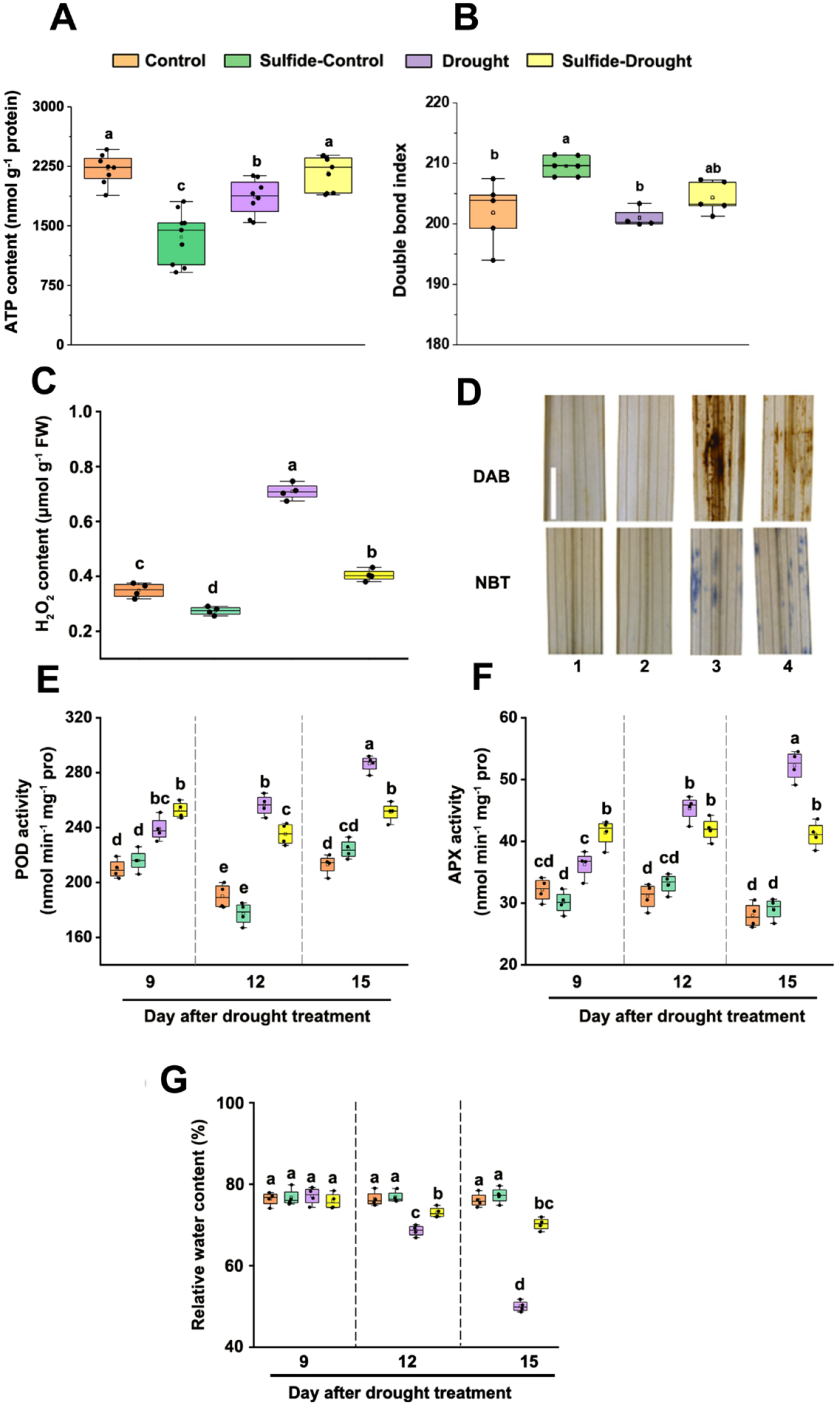
Effect of exogenous application of NaHS on ATP content, fatty acid double bond index (DBI), redox homeostasis, and water loss of rice leaves under drought stress. (A) Determination of ATP content (*n*=9); (B) DBI (*n*=5) calculated on the basis of the fatty acid composition; (C) H_2_O_2_ content (*n*=4); (D) histochemical analyses for H_2_O_2_ (DAB staining) and O_2_^−^ (NBT staining); (E) POD activity (*n*=4); (F) APX activity (*n*=4); (G) relative water content (*n*=3). All experiments were performed using 25-day-old rice plants pre-treated or not with 50 μM NaHS for 10 d and subjected to drought or normal irrigation for 15 d (A–D) or 9, 12, and 15 d (E–G). Data are means ±SD. The same letters indicate no significant differences at *P*<0.05 (Duncan’s multiple range tests). Scale bar=4 cm.

The analysis described above showed that the highest enrichment of the most persulfidated proteins under drought conditions corresponds to enzymes of the TCA cycle and other related pathways such as fatty acid degradation (Supplementary Fig. S3; Table 1), which could indicate the role of persulfidation in metabolic reprogramming. The TCA cycle is of central importance not only in the biosynthesis of metabolites but also in the generation of energy. Keeping in mind that the role of mitochondrial metabolism in water stress has been reported (Skirycz *et al.*, 2010), we analyzed the levels of total ATP under drought stress in rice tissues treated or not with sulfide. We observed a change in ATP content under drought stress, that was reversed to the control levels in samples pre-treated with sulfide under drought stress, which might indicate that persulfidation of proteins related to the TCA cycle regulates energy production (Fig. 3A).

Several enzymes from the fatty acid degradation pathway were also more persulfidated under drought stress, such as two 3-ketoacyl-CoA thiolases, an acetyl-CoA acetyltransferase, and a long chain acyl-CoA synthetase. The fatty acid composition analysis showed that total fatty acids did not change substantially after NaHS treatment or drought stress, although some differences were observed in the level of oleic (18:1), linoleic (18:2), and linolenic (18:3) acids when compared with control conditions, that correlated with significantly different DBIs. The level of 18:3 increased significantly with a concomitant decrease in 18:1 and 18:2 acids (Supplementary Table S3) as well as a significant increase of the DBI in NaHS-irrigated plants (Fig. 3B), which is indicative of an effect of sulfide on the omega-3 desaturases. When drought was imposed after sulfide pre-treatment, the DBI approached the level observed under control conditions.

Moreover, as indicated by the GO enrichment analysis, many identified proteins in the proteomic analysis involved in cellular redox regulation changed their level of persulfidation depending on the conditions, more persulfidated in control and less persulfidated in response to drought stress (Table 1). Thus, the proteomic analysis presented above suggested that the antioxidant system of rice may be regulated by persulfidation in response to drought stress. Under our experimental conditions, drought stress triggered ROS accumulation, as indicated by the H_2_O_2_ content (Fig. 3C) and histochemical analyses of H_2_O_2_ (DAB staining) and O_2_^−^ (NBT staining) in rice leaves (Fig. 3D), while NaHS pre-treatment reduced the drought-induced ROS accumulation, indicating that H_2_S plays a role in regulating redox homeostasis in rice leaves under drought. To further decipher the mechanism of H_2_S pre-treatment for alleviation of drought stress, we determined related antioxidant enzymatic activities, mainly PODs, which presented reduced persulfidation levels under drought stress (Supplementary Table S2). As shown in Fig. 3E and F, the activities of total PODs and APX in leaves exhibited an increasing trend during drought stress treatment, which is consistent with their role as antioxidant defenses that reduce the progressive accumulation of ROS observed at the end of the drought treatment (15 d) (Fig. 3C, D). However, since NaHS pre-treatment reduced ROS accumulation, increased antioxidant defenses were no longer needed and, consequently, decreased POD and APX activities were measured (Fig. 3E, F). These results suggest that sulfide pre-treatment also alleviated rice drought stress by regulating the antioxidant system and modulating ROS homeostasis.

### Sulfide regulates cellular water homeostasis and water transport

GO enrichment analysis of proteins with higher levels of persulfidation under drought also showed a high enrichment in the categories of cellular water homeostasis and ion transmembrane transport. Accordingly, we analyzed the effect of NaHS pre-treatment on the RWC of rice leaves subjected to drought stress (Fig. 3G). The results showed that during drought stress treatment, the RWC of rice leaves decreased, while NaHS pre-treatment significantly attenuated the water loss compared with the loss observed in the absence of this pre-treatment. This evidence hints that adaptation responses to drought stress in rice plants may include the regulation of sulfide-mediated water transport.

Subsequently, we evaluated the effect of NaHS treatment on water transport activity of rice protoplasts using a stopped-flow approach. This technique has been successfully applied following changes in the scattered light in both lipid and membrane vesicles, but also in protoplasts of whole yeast cells (Chen *et al.*, 1988; Gerbeau *et al.*, 2002; Bertl and Kaldenhoff, 2007; Otto *et al.*, 2010; Ding *et al.*, 2019). However, in plant cells, water transport can be followed by changes in chlorophyll fluorescence (Chen and Knutson, 1988; Chen *et al.*, 1988; Wachlmayr *et al.*, 2022). In this particular scenario, the net emitted light is the result of the chlorophyll fluorescence quenching, that increases when the concentration of the inside fluorophore (chlorophyll) in the shrunken protoplasts increases (or decreases when protoplasts expand).

**Fig. 4.**
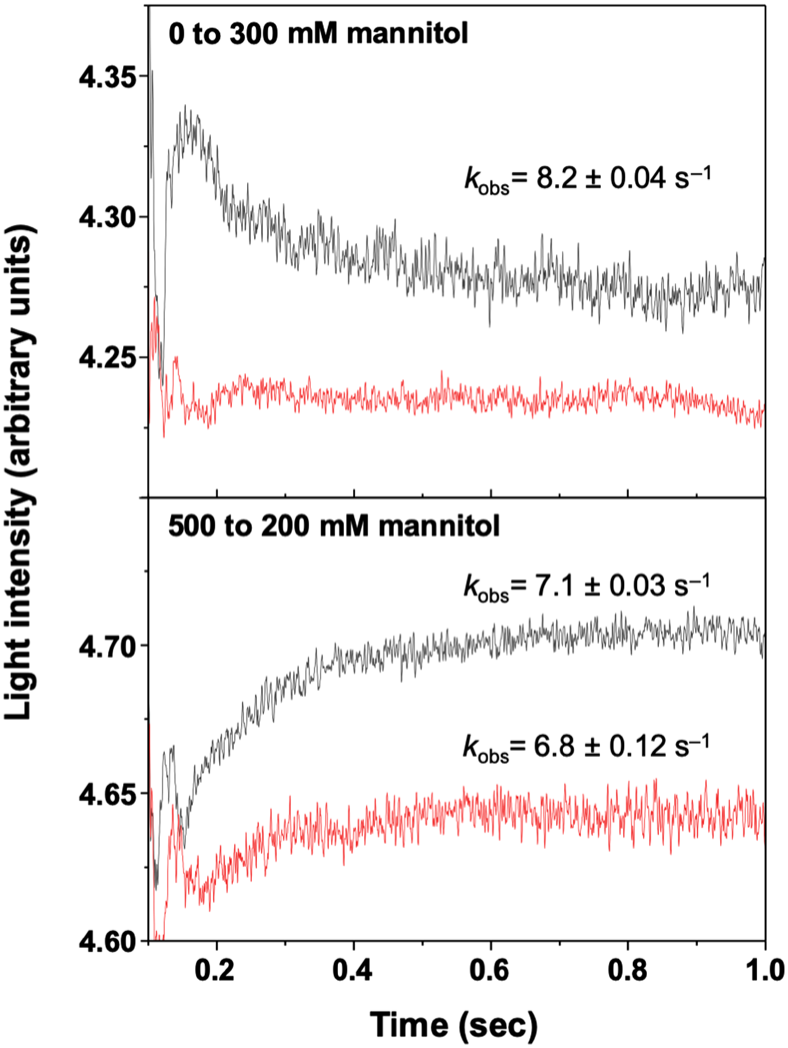
NaHS treatment inhibited water transport activity of rice protoplasts. Stopped-flow measurements of the osmotic water permeability of untreated (black traces) and 10 μM NaHS-treated (red traces) rice protoplasts. The protoplasts were suspended in the absence of mannitol and then submitted in a stopped-flow apparatus to 300 mM mannitol (upper panel), or suspended in 500 mM mannitol and then submitted to 200 mM (lower panel), and changes in the measured light intensity concomitant with volume change were monitored. Each kinetic trace was an average of 8–10 individual repeats, and the calculated *k*_obs_ values (mean ±SD) are shown.

Thus, after quickly exposing untreated protoplasts to a shift from hypotonic to hypertonic buffer (Fig. 4, upper panel), and vice versa from hypertonic to hypotonic buffer (Fig. 4, lower panel), the time course of the change in protoplast volume was recorded by measuring the intensity of the emitted light. The application of a hypertonic gradient results in shrinkage of protoplasts due to the water efflux, leading to a monophasic decrease in the fluorescence signal emitted (*k*_obs_=8.2 ± 0.04 s^–1^; Fig. 4, upper panel). The value of *k*_obs_ estimated here is of the same order of magnitude as previously found in other plant systems, including water influx by plant aquaporins in yeast whole-cell protoplasts (Gerbeau *et al.*, 2002; Bertl and Kaldenhoff, 2007; Sommer *et al.*, 2007; Otto *et al.*, 2010; Leitao *et al.*, 2012). However, when protoplasts were previously treated with NaHS, no changes in light intensity were observed, due to the inhibition of the water efflux and the consequent changes in protoplast volume (Fig. 4, upper panel). In contrast, the application of a hypotonic gradient resulted in an increase in the intensity of the light emitted due to water influx (*k*_obs_=7.1 ± 0.03 s^–1^). In this case, NaHS treatment did not fully abolish the water influx, but induced a drastic decrease in the amplitude of the signal (~75%), although maintaining a similar *k*_obs_ value (6.8 ± 0.12 s^–1^), indicating that most of the water permeability of the protoplasts was reduced (Fig. 4, lower panel).

### Sulfide-dependent aquaporin persulfidation

**Fig. 5.**
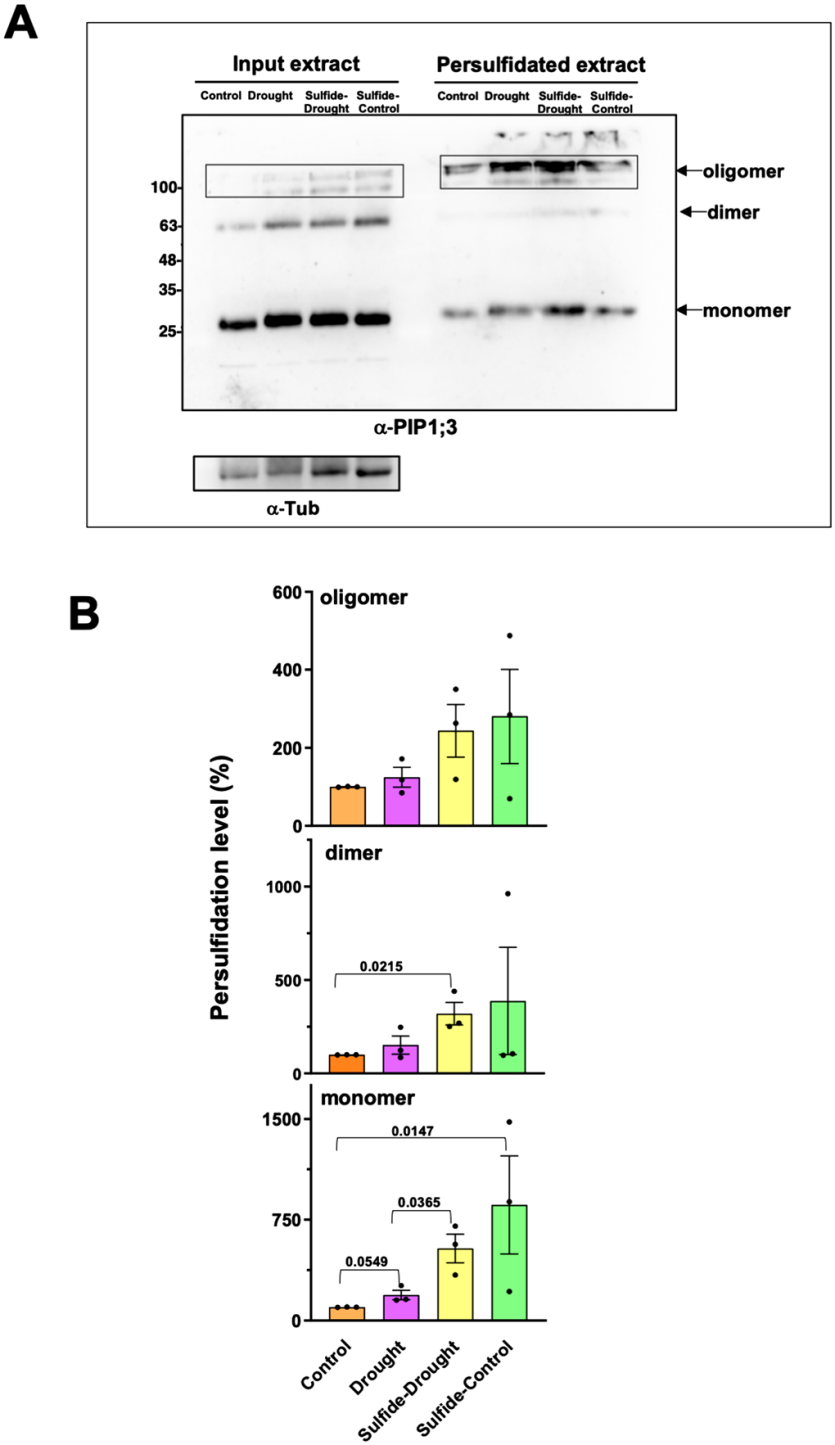
Persulfidation of aquaporins in response to drought stress. (A) Immunoblot analysis of persulfidated aquaporins. Protein extracts were prepared from rice leaves treated as described in Supplementary Fig. S1 and divided into two batches; one was used directly for immunoblotting (input extract) and the other was first subjected to the tag-switch method (persulfidated extract) as described in the Materials and methods. The protein extracts were subjected to SDS–PAGE and immunoblot analysis with anti-PIP1;3 antibodies. Anti-Tub antibodies were used as the protein loading control of the input extract. Arrows indicate the monomeric, dimeric, and oligomeric forms of aquaporins. A representative image is shown. (B) Quantification of the level of persulfidation of each aquaporin form. For each condition, the immunodetected proteins were quantified and the level of each persulfidated protein was referred to its level in the input extract, in turn, relative to the tubulin protein level. The value of 100% was assigned to the level of persulfidation in control conditions. Data (mean ±SE) are from three independent experiments and were analyzed statistically by unpaired *t*-test. The *P*-values are shown.

As described above, eight aquaporins are enriched in the category of cellular water homeostasis and, more importantly, all of them were shown to be more persulfidated in response to drought stress than under the control condition (Table 2). Thus, the observed sulfide regulation of water transport of rice protoplasts could be indicative of the functional regulation of aquaporins by sulfide-dependent persulfidation in response to drought. To address this, we performed an in-gel detection of persulfidation by immunoblotting with anti-PIP1;3 antibodies that also detect other PIP proteins. Immunoblots showed a protein band of ~25 kDa, with roughly the expected size for the monomeric form of aquaporins, and additional bands that corresponded to the dimeric and oligomeric forms (Fig. 5A). This protein profile is similar to the one observed in other studies (Ohshima *et al.*, 2001; Otto *et al.*, 2010). Quantification of the persulfidation level of each aquaporin form was done relative to the amount of each one in the different samples in order to differentiate any possible increase of protein amount after drought stress from the effect on the persulfidation level (Fig. 5B). In this way, a clear increase in the level of persulfidation of all aquaporin forms in response to drought stress was detected, which was further increased when exogenous NaHS pre-treatment was performed. However, statistical analysis of the data showed that the induction of the persulfidation level under drought was statistically significant in the case of the monomeric forms. We observed a 2- and 5-fold increase in persulfidation level under drought conditions and drought after sulfide pre-treatment, respectively, when compared with control conditions. Collectively, our findings suggest that sulfide regulates water transport activity, which is probably associated with the persulfidation of aquaporins under drought stress.

## Discussion

An increasing number of studies have revealed H_2_S as a signaling molecule that regulates essential plant processes, including adaptation responses (Gotor *et al.*, 2019; Aroca *et al.*, 2021b). Nevertheless, knowledge of the underlying molecular mechanism of sulfide signaling is still scarce and is starting to be deciphered only in the model plant *A. thaliana*. In this plant, the post-translational modification of persulfidation has been demonstrated that results in changes in protein function. Proteomic analyses performed in Arabidopsis tissues have revealed a catalog of proteins susceptible to persulfidation (Aroca *et al.*, 2015, 2017a; Laureano-Marin *et al.*, 2020; Jurado-Flores *et al.*, 2021), although only a few proteins have been thoroughly studied as targets for persulfidation. The precise mechanism of H_2_S signaling is mainly known in the regulation of autophagy and ABA-dependent stomatal closure, both processes in Arabidopsis (Aroca *et al.*, 2021b; Gotor *et al.*, 2022). However, at present, scanty information about protein persulfidation exists in crop species, and the precise mechanism of the sulfide regulation of stress responses that would be useful to improve crop resilience is completely unknown.

In our proteomic study, we describe the first persulfidation proteome in a crop and reveal that 3443 different proteins are susceptible to persulfidation in rice. Similar to Arabidopsis, persulfidated proteins are widely present in rice cells, participating in a broad range of biological functions and are involved in different metabolic pathways, all suggesting that persulfidation is a post-translation modification with a high impact also in cereal plants. Furthermore, the comparative proteomic approach resulted in the first identification of specific proteins with quantitatively different levels of persulfidation under drought stress, which could be specific targets involved in the sulfide signaling of drought responses in rice.

The role of H_2_S in improving tolerance to drought has been studied in different plant species (Jin *et al.*, 2011; Ma *et al.*, 2016). During drought stress, the overproduction of ROS causes the disruption of cellular redox homeostasis, which severely impedes plant growth (Miller *et al.*, 2010). In rice in particular, H_2_S promotion of drought tolerance by the reduction of ROS production and mitigation of oxidative stress has been shown (H. Zhou *et al.*, 2020, 2021), although the underlying mechanism was not demonstrated. We also observed that H_2_O_2_ accumulation under drought was significantly reduced by previous sulfide treatments, and further identified a high proportion of proteins involved in maintaining cellular redox homeostasis that showed distinct persulfidation levels under control and drought conditions (Fig. 2). Furthermore, in the samples pre-treated with sulfide, ROS production and peroxidase activities decreased under drought stress, which could indicate that the plants treated with sulfide seemed to be affected by a milder stress than the untreated plants. Therefore, sulfide pre-treatment before plants are subjected to drought stress appears to induce a physiological state in them that allows a more efficient response to drought, and plants require no further activation of their defensive responses. This role of sulfide resembles the activation of a priming mechanism, which has already been described, although the molecular mechanism remains elusive (Mauch-Mani *et al.*, 2017). This assumption is consistent with all drought stress markers measured, such as phenotypic traits, drought-responsive gene transcription levels, ABA accumulation, and RWCs, where a significantly reduced impact of drought stress was observed in sulfide-pre-treated rice plants. Reactive oxygen, nitrogen, and sulfur species have been suggested as priming agents based on their essential role in plant stress acclimation (Antoniou *et al.*, 2016), and, recently, nitric oxide and hydrogen sulfide (NOSH)-releasing synthetic compounds have been proposed as novel priming agents against drought stress in *Medicago sativa* (Antoniou *et al.*, 2020). Further research will be required to ascertain the role of H_2_S as a priming agent and if the molecular mechanism involved is the persulfidation of specific targets.

When faced with water limitation, plants actively reprogram their metabolism to maintain growth on the one hand and ensure survival on the other (Claeys and Inze, 2013). The proteomic data of this study reveal a clear prioritization of the persulfidation of proteins involved in the TCA cycle and related pathways to achieve metabolic reprogramming under drought. Drought-induced metabolic reprogramming has been shown to increase intermediates of the TCA cycle (Pires *et al.*, 2016), which is related to their utilization in mitochondrial metabolism. Our findings suggest that H_2_S signaling in this pathway is a primary response to drought in rice to regulate energy production. In accordance, H_2_S has been shown to be a regulator of energy production in mitochondria to delay drought stress-induced senescence in Arabidopsis (Jin *et al.*, 2018).

Modification of fatty acid levels and the degree of desaturation are included in the battery of plant responses to multiple abiotic stresses for acclimation (Zhang *et al.*, 2005). In our study, we do not observe a significant decrease in the proportion of unsaturated fatty acids under drought stress, as previously observed in different plant systems (Hamrouni *et al.*, 2001; Sanchez-Martin *et al.*, 2018). Interestingly, sulfide pre-treatment results in an increase in the DBI, which has been associated with tolerance to dehydration by maintaining membrane fluidity (Hoekstra *et al.*, 2001; Sanchez-Martin *et al.*, 2018). These results (Fig. 3) further reinforce the role of sulfide as an inducer of either a higher stress threshold or faster drought sensing to trigger a response.

Maintaining leaf water content is critical, and its regulation constitutes an essential plant response for tolerance to drought stress. Of relevance are aquaporins that form a large family of integral membrane proteins that facilitate the transport of water and small neutral molecules across biological membranes (Maurel *et al.*, 2015). Our proteomic study revealed that six aquaporins localized in the plasma membrane (PIPs) and two others in the tonoplast (TIPs) are significantly persulfidated under drought stress. Interestingly, all identified PIPs and TIPs have been proven to positively regulate rice growth, development, and tolerance to osmotic stress. Expression of PIP1-1 increases salt resistance (Liu *et al.*, 2013); PIP1-2 participates in sucrose transport under elevated CO_2_ concentration (Xu *et al.*, 2019); expression of PIP1-3 promotes seed germination under water stress (Liu *et al.*, 2007); PIP2-1 confers root growth under drought stress (Ding *et al.*, 2019); PIP2-2 is relevant in the tolerance to drought (Bai *et al.*, 2021); and PIP2-7 enhances the transpiration rate and low temperature tolerance (Li *et al.*, 2008b). Regarding the tonoplast forms, TIP1-1 and TIP1-2 are strongly induced by salt and drought stress (Li *et al.*, 2008a; Balasaheb Karle *et al.*, 2020).

Our findings highlight that aquaporins are significantly more persulfidated upon drought stress in rice and suggest that sulfide regulation of water transport is a primary adaptation response to drought in rice. Indeed, we have demonstrated that the water efflux transport activity in rice protoplasts is negatively regulated by sulfide, and that this regulation may correlate to an *in vivo* increase in the level of persulfidation of aquaporins when rice plants are subjected to drought stress, even without providing an exogenous source of sulfide (Fig. 4). Therefore, we can conclude that aquaporin persulfidation could be linked to adaptation to drought in rice. Interestingly, H_2_O_2_ is also a potential substrate for aquaporins (Rhee *et al.*, 2017), which can also regulate their subcellular redistribution in roots (Wudick *et al.*, 2015). Exogenous NO treatment in rice also induces the expression of several OsPIPs in germinating seeds under water stress (Liu *et al.*, 2007), and H_2_S treatment in strawberry plants subjected to heat shock is accompanied by reduced levels of H_2_O_2_ that coincided with increased expression of aquaporins (Christou *et al.*, 2014); however, we did not observe increased expression of aquaporins in leaves treated or not with NaHS under drought (Fig. 5A). Nevertheless, sulfide regulation of the H_2_O_2_ transport capacity should not be ruled out as a mechanism of tolerance to drought. As a matter of fact, human aquaporin-8 gating has been shown to be mediated by persulfidation of Cys53, thus controlling H_2_O_2_ transport (Bestetti *et al.*, 2018). In addition, sulfenylation of Cys191 of human aquaporin-1 increased the free energy barrier for H_2_O_2_ and NO_2_ permeation (Yusupov *et al.*, 2019), and therefore persulfidation could also compete with this oxidative post-translational modification and be part of a complex regulation scheme in response to oxidative stress.

Our proteomic study has revealed a new post-translational modification of rice aquaporins, which are already known to exhibit other modifications such as *S*-nitrosylation and *S*-sulfenylation (Maurel *et al.*, 2015; Mukherjee *et al.*, 2024). Persulfidation is a new regulatory mechanism of aquaporins, which increased significantly under drought stress. Recently, several aquaporins such as PIP1-2, PIP2-1, and PIP2-2 have been also reported to be persulfidated in Arabidopsis under non-photorespiratory conditions (Garcia-Calderon *et al.*, 2023); however, an additional proteomic study showed no cross-links with water transport activity and persulfidated aquaporins in response to drought in Arabidopsis (Jurado-Flores *et al.*, 2023a), pointing out that plant sulfide-dependent responses are probably species specific. In connection with this, rice has more transpiration and, therefore, is more sensitive to water deficit, which is effectively combated by the water transport provided by aquaporins (Ding *et al.*, 2019). Therefore, regulation of water transport activity connected to the persulfidation of aquaporins could be among the relevant drought responses, but specifically in rice.

**Fig. 6.**
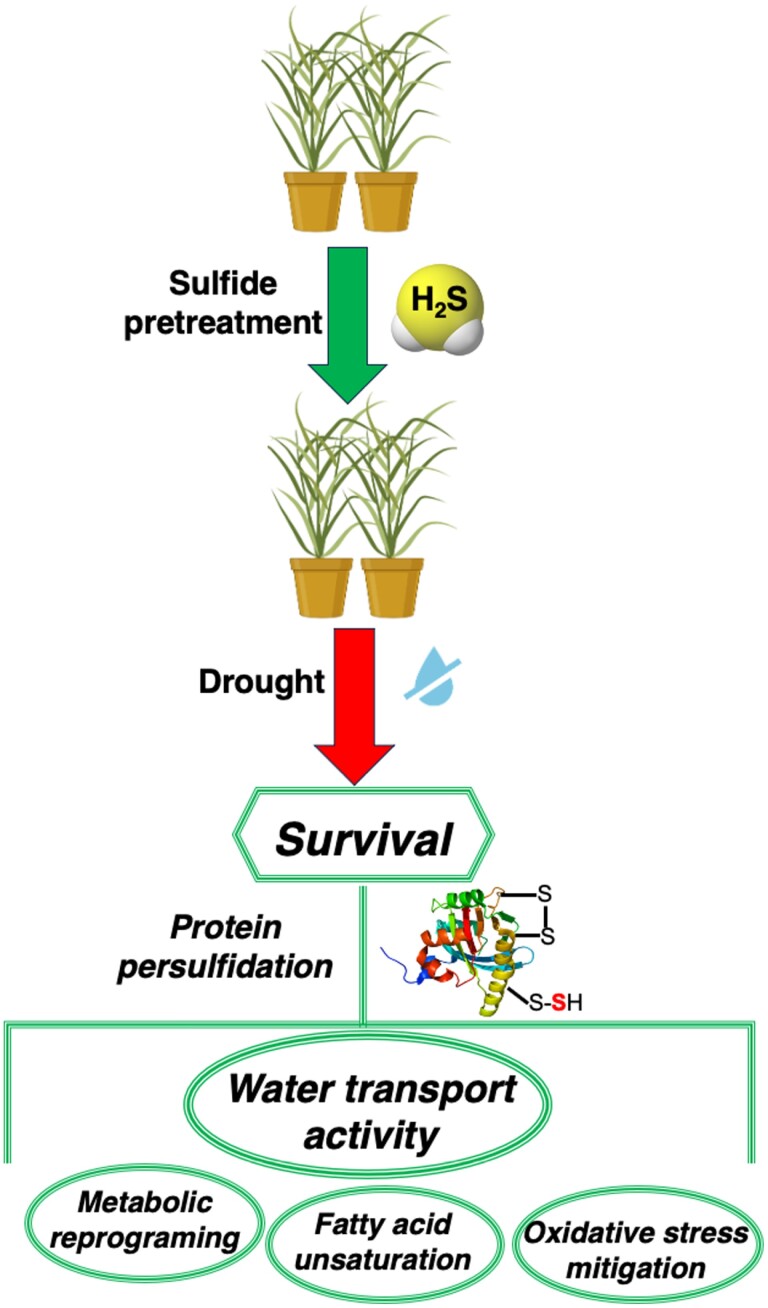
Graphical outline of the impact of H_2_S pre-treatment on rice under drought.

In conclusion, we provide the first persulfidation proteome in rice tissues, revealing that protein persulfidation is a widely distributed post-translational modification involved in numerous biological processes. Comparative and quantitative proteomic analyses have also demonstrated the impact of persulfidation on a variety of specific responses to drought (Fig. 6), including metabolic reprogramming for energy generation, increased fatty acid unsaturation associated with membrane fluidity, and mitigation of oxidative stress. Remarkably, water transport associated with aquaporin persulfidation seems to be an important drought response specifically in rice plants.

## Supplementary data

The following supplementary data are available at *JXB* online.

Dataset S1. Total persulfidated proteins identified in this study.

Dataset S2. Functional annotation by GO biological processes and KEGG pathway of total persulfidated proteins.

Dataset S3. Identified proteins only present or more persulfidated under drought stress conditions.

Dataset S4. Identified proteins only present or more persulfidated under control conditions.

Table S1. Primers used in this study.

Table S2. Proteins classified within the GO_biological process term ‘hydrogen peroxide catabolic process’.

Table S3. Fatty acid compositions (mol%) in rice plants under control and drought stress.

Fig. S1. Workflow of treatments in rice plants.

Fig. S2. Representative image of isolated protoplasts.

Fig. S3. Schematic representation of the enriched biological pathways and processes containing the significantly most persulfidated proteins in drought samples.

erae249_suppl_Supplementary_Datasets_S1-S4

erae249_suppl_Supplementary_Figures_S1-S3

erae249_suppl_Supplementary_Tables_S1-S3

## Data Availability

The MS proteomic data have been deposited to the ProteomeXchange Consortium via the PRIDE partner repository with the identifier PXD036637.
