# Supplementary material for: Analysis of sulfide signaling in rice highlights specific drought responses
Source: J Exp Bot. 2024 May 29;75(16):5130–45. doi: 10.1093/jxb/erae249 (PMC11349868; doi:10.1093/jxb/erae249)
Supplement: erae249_suppl_Supplementary_Figures_S1-S3 [file erae249_suppl_supplementary_figures_s1-s3.pdf]

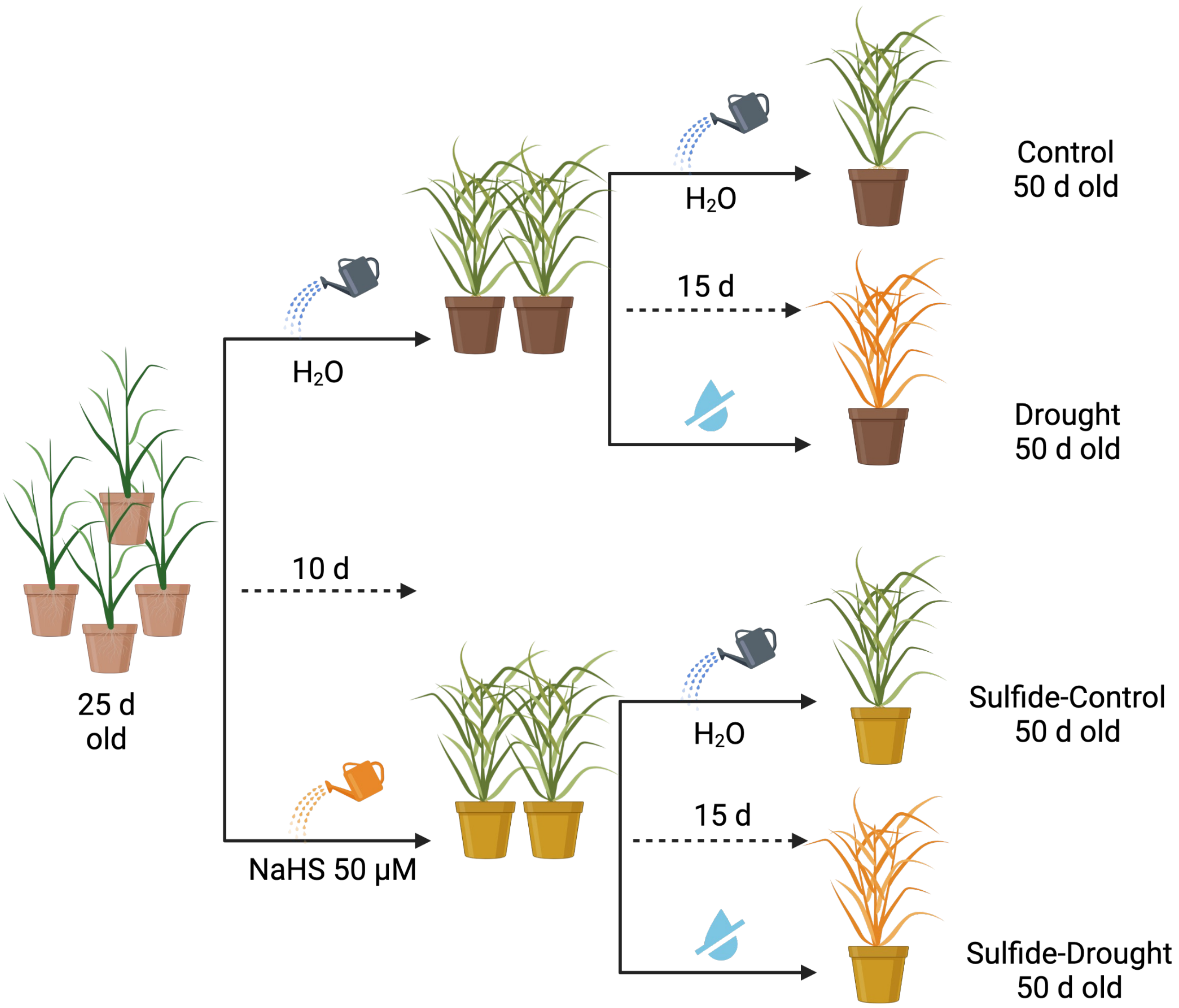

Figure S1 Workflow of the treatments in rice plants. 25-day-old plants grown in soil under physiological conditions were divided into two batches; one batch was irrigated with water and the other with 50 µM NaHS for 10 additional days. After this period, each batch was subsequently divided into two new batches and subjected to water irrigation or drought for other 15 additional days. At the end of the full treatment, 4 different plant samples were obtained and named as shown in the figure (control, drought, sulfide-control and sulfide-drought)

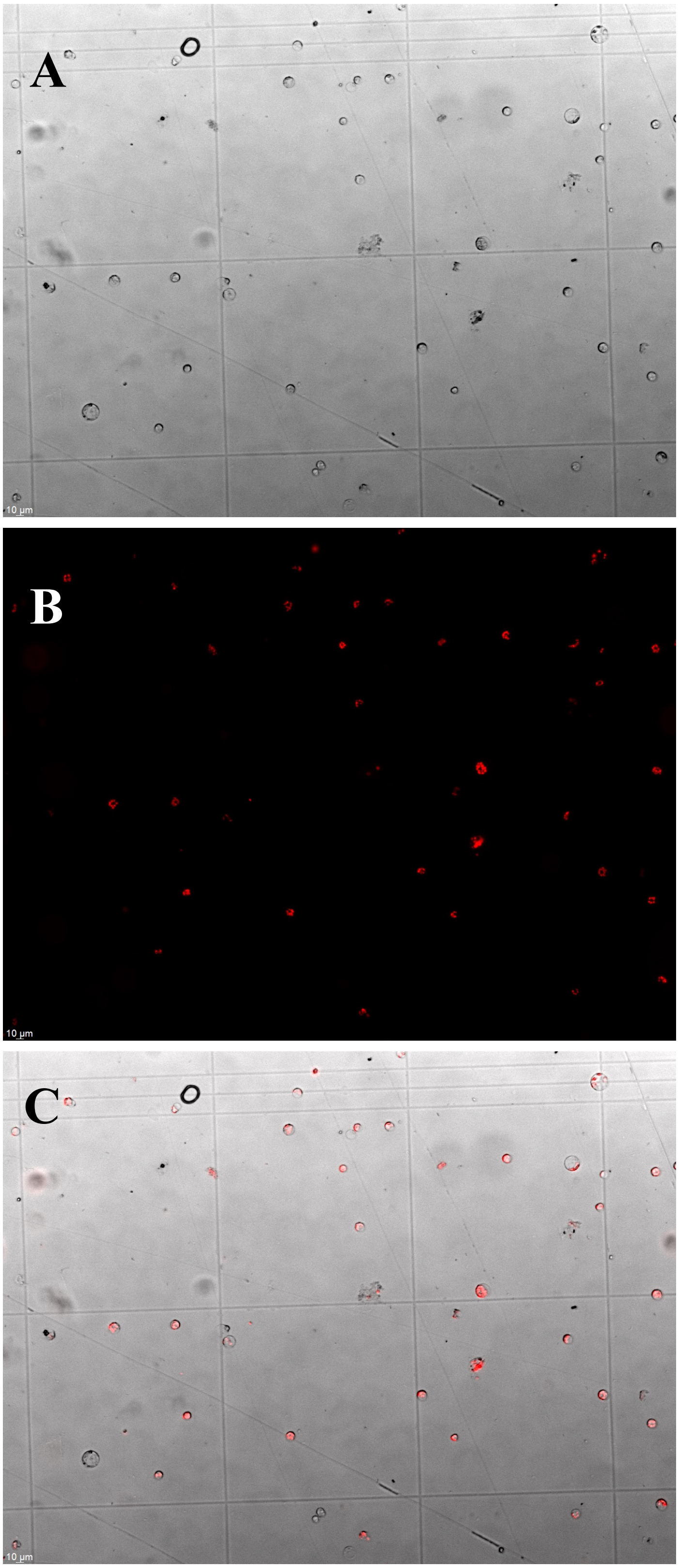

Figure S2. Representative image of isolated protoplasts. A, Bright field image. B, Fluorescent image. C, Merged image of A and B.

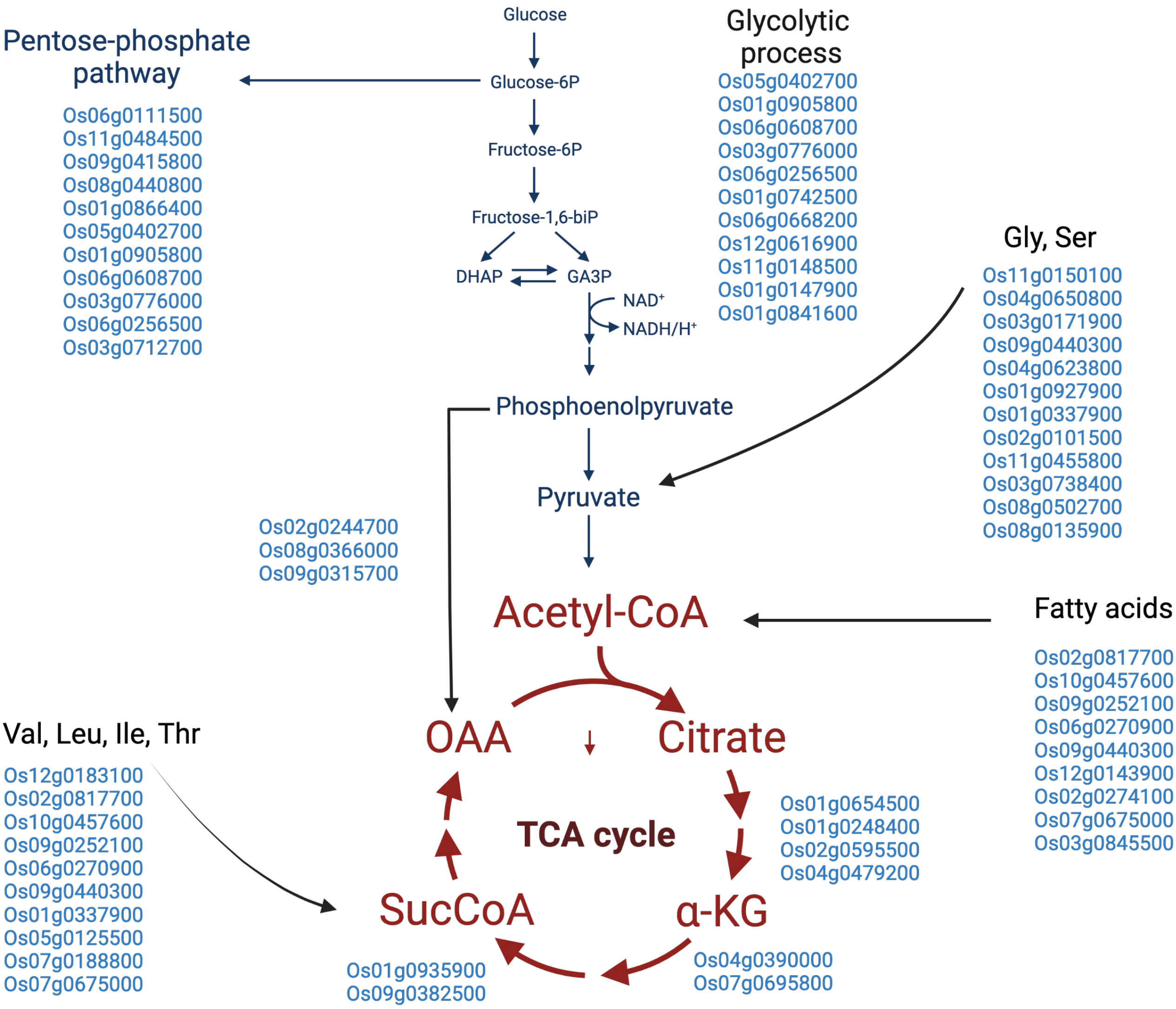

Figure S3 Schematic representation of the enriched biological pathways and processes containing the significantly most persulfidated proteins in drought samples. Locus_ID of the identified proteins annotated in each GO_Biological or KEGG Pathway are shown.
