# Supplementary material for: Analysis of sulfide signaling in rice highlights specific drought responses
Source: J Exp Bot. 2024 May 29;75(16):5130–45. doi: 10.1093/jxb/erae249 (PMC11349868; doi:10.1093/jxb/erae249)
Supplement: erae249_suppl_Supplementary_Tables_S1-S3 [file erae249_suppl_supplementary_tables_s1-s3.pdf]

Table S1 Primers used in this study

Primer name	Primer Sequence (5'-3')
OsNCED1-L	GATCAAGAAGCCGTACCTCAA
OsNCED1-R	CCACGTAGTTCTCGGTGATG
OsPP2C30-L	GCTATTTGAAGCCGTACGTGATA
OsPP2C30-R	TGGCCAGGATGAGGAACT
OsUBQ5-L	CATGGACTGGTTAAATCAATCGTCA
OsUBQ5-R	TACCATATACCACGACCGTCAAAA
OsACTIN1-L	CAACACCCCTGCTATGTACG
OsACTIN1-R	CATCACCAGAGTCCAACACAA

Table S2 Proteins classified within the GO_biological process term "hydrogen peroxide catabolic process".

Locus	Gene Name
Os03g0285700	L-Ascorbate peroxidase 1, cytosolic-like (LOC4332474)
Os06g0306300	Peroxidase (Os06g0306300)
Os07g0157600)	Peroxidase (Os07g0157600)
Os10g0109300)	Peroxidase (Os10g0109300)
Os01g0962700	Cationic peroxidase SPC4(LOC4324554)
Os01g0963000	Cationic peroxidase SPC4(LOC4324556)
Os04g0688600	Cationic peroxidase SPC4(LOC4337482)
Os01g0327400	Peroxidase 1(LOC4326970)
Os01g0326000	Peroxidase 1(LOC4327001)
Os01g0294700	Peroxidase 2(LOC4326716)
Os05g0134400	Peroxidase 2(LOC4337725)
Os08g0113000	Peroxidase 47(LOC4344496)
Os03g0339300	Peroxidase 70(LOC4332782)
Os02g0236600	Peroxidase P7(LOC4328832)
Os02g0236800	Peroxidase P7(LOC4328833)
Os02g0240300	Peroxidase P7(LOC4328842)
Os02g0553200	Probable L-ascorbate peroxidase 8, chloroplastic(LOC4329643)
Os12g0188700	Thioredoxin M5, chloroplastic-like (LOC9270622)

Table S3 Fatty acid compositions (mol%) in rice plants under control and drought stress.

Component	Watering condition			
	Control	NaHS	Drought	NaHS+Drought
**C16:0 Palmitic**	19.43 ± 0.52	18.80 ± 0.35	20.07 ± 1.06	19.09 ± 0.75
**C16:1 Palmitoleic**	0.84 ± 0.08	0.91 ± 0.03	1.01 ± 0.06	0.91 ± 0.05
**C18:0 Stearic**	3.30 ± 0.27	2.91 ± 0.11	3.20 ± 0.89	2.69 ± 0.28
**C18:1 Oleic**	2.63 ± 0.23	1.41 ± 0.05	1.51 ± 0.21	1.42 ± 0.04
**C18:2 Linoleic**	14.01 ± 0.20	13.31 ± 0.25	16.15 ± 1.00	16.86 ± 0.88
**C18:3 Linolenic**	57.17 ± 1.35	60.23 ± 0.61	55.14 ± 1.69	56.46 ± 0.72

Data are means (n = 6) ± standard deviation.
